# Synergistic effect of fruit–seed mixed juice on inhibition of angiotensin I-converting enzyme and activation of NO production in EA.hy926 cells

**DOI:** 10.1007/s10068-018-0512-0

**Published:** 2018-11-23

**Authors:** Hye-Jung Park, Ji-Youn Kim, Hee Sook Kim, Sang-Hyeon Lee, Jeong Su Jang, Mun Hyon Lee

**Affiliations:** 1 Food Research Center, Angel Co., Ltd., Busan, 46988 Korea; 2 Major in Pharmaceutical Engineering, Division of Bioindustry, College of Medical and Life Sciences, Silla University, Busan, 46958 Korea

**Keywords:** Angiotensin I-converting enzyme, Hypertension, Fruit–seed mixed juice, Synergistic effect, Antioxidant

## Abstract

Commonly consumed fruit juices possess low inhibitory activity of angiotensin I-converting enzyme (ACE), which plays central role in elevation of blood pressure. The ACE inhibitory activity of fruit–seed mixed juice may be improved via synergistic interactions. In this study, the investigated synergistic, additive, and antagonistic effects of fruit–seed combination on ACE inhibition were investigated. Thirteen fruits and 15 seeds including legumes, nuts, and cereals were combined in pairs; pear-hemp seed-pumpkin seed juice (3-mixed juice) displayed the highest ACE inhibition resulting from synergistic interactions. Additionally, nitric oxide production in human endothelial cells was promoted by 3-mixed juice. Three-mixed juice showed antioxidant activities such as DNA protective, DPPH radical scavenging, and reducing effects. These results suggested that combinations of different food categories are beneficial for improving biological functions such as vascular health. Three-mixed juice, which shows high ACE inhibitory activity, may be useful as an anti-hypertensive agent and for treating hypertension.

## Introduction

Cardiovascular diseases (CVD), which are among the most common adult diseases, are leading causes of death among Organisation for Economic Co-operation and Development (OECD) member states. Jimsheena and Gowda (2011) reported that CVDs was caused by consumers’ reduced physical activity and increasing consumption of high-energy foods. Among other ordinary CVDs, hypertension can cause metabolic disorders including obesity, prediabetes, and atherosclerosis (Tavares et al., 2012). Increased blood pressure (BP) can be controlled by antihypertensive drugs. Synthetic drugs, however, have side effects such as cough, skin rash, or angioneurotic edema (Riccioni et al., 2009; Wijesekara and Kim, 2010). As the side effects of many different synthetic drugs have been determined and published widely, research on the bioactive function of natural foods has recently gained attention.

Natural foods lower BP in various manners and are divided into those that induce the release of nitric oxide (NO) in blood vessels, act as diuretics, and inhibit the renin-angiotensin system (RAS). Particularly, the RAS is a central system that regulates BP; renin released from the kidney breaks down angiotensinogen into angiotensin I, and angiotensin I is converted to angiotensin II as the C-terminal dipeptide His-Leu is removed by the angiotensin I-converting enzyme (ACE) (Lavoie and Sigmund, 2003). Angiotensin II increases BP through renal reabsorption of sodium and water directly and indirectly by increasing the secretion of aldosterone, arginine vasopressin. In addition, ACE removes and renders inactive the C-terminal dipeptide Phe-Arg from bradykinin and kallidin, which dilate blood vessels (Yang et al., 1970). Bradykinin causes blood vessels to dilate by combining with β-receptors and converts l-arginine into NO by increasing the Ca^2+^ levels and stimulating nitric oxide synthases. Hence, ACE raises BP by inhibiting NO production indirectly. Most antihypertensive drugs, such as captopril, enalapril, and fosinopril exert antihypertensive effects by inhibiting ACE, which turns angiotensin I into angiotensin II (Sweitzer, 2003). A variety of food-based ACE inhibitors have been discovered, and most are made up of peptides originating from proteins such as milk, meat, egg, and fish (Iwaniak et al., 2014). Additionally, peptides produced by soybean, sunflower, flaxseed, rice, and corn, anthocyanins originating from apple peels, and polyphenols such as flavan-3-ols are widely known as plant-based inhibitors (Actis-Goretta et al., 2006). Fruit-based inhibitors, however, show a relatively lower level of ACE inhibition than seed-based inhibitors, but few research and development studies have been conducted. In this regard, by examining the synergistic anti-hypertensive effect of fruit–seed mixed juice produced by adding seeds to fruits, which are often consumed as beverages, this study aims to develop a fruit–seed mixed juice beverage to improve cardiovascular health and function.

## Materials and methods

### Materials and sample pretreatment

Fruits (apple, aronia, banana, grape, grapefruit, melon, nectarine, orange, oriental melon, peach, pear, pinapple, and plum) and seeds (black soybean, common buckwheat, evening primrose seed, hemp seed, job’s tear, lentils, mung bean, oat, peanut, perilla seed, pumpkin seed, safflower seed, sesame seed, sunflower seed, and tartary buckwheat) used in this experiment were purchased from a farmers’ market (Busan, Korea) in March 2017. The purchased fruits and seeds were washed with water and then made into extracts using a lower-speed juice extractor at 82 rpm (Angelia 8000, Angel Co., Ltd., Busan, Korea). Fruits and seeds were mixed in extracts at a ratio of 7–3, and the extracts were diluted by fivefold in distilled water for 2 h at ambient temperature and then used as a sample.

### Chemicals

Gallic acid, naringin, procyanidin B2, 3-(4,5-dimethylthiazol-2-yl)-2,5-diphenyltetrazolium bromide (MTT) and 2,2-diphenyl-1-picrylhydrazyl (DPPH) were supplied by Sigma-Aldrich (St. Louis, MO, USA). Grape seeds oligomeric proanthocyanidins was supplied by United States Pharmacopeial Convention (Rockville, MD, USA). Dulbecco’s modified Eagle’s medium (DMED) and fetal bovine serum (FBS) were supplied by Hyclone Laboratories (Logan, UT, USA).

### Determination of the ACE inhibitory activity

The ACE inhibitory activity was analyzed according to a modified standard process (Cushman and Cheung, 1971). To determine ACE inhibitory activity, 100 µL of 0.1 M sodium borate buffer (pH 8.3) and 50 µL of crude ACE were mixed in solution, followed by addition of 50 µL of the HHL (N-hippuryl-histidyl-leucine). This solution was reacted for 30 min at 37 °C. After stopping the reaction by adding 250 µL of 1 N HCl, 1.5 mL of ethyl acetate was added. This mixture was agitated for 15 s and centrifuged (2012×*g*, 5 min, 4 °C); 1 mL of the supernatant was obtained. After completely drying the supernatant followed by agitation with 1 mL distilled water, its absorbance was measured with a spectrophotometer (Multiskan GO, Thermo Scientific, Vantaa, Finland) at 228 nm.

### Analysis of basic composition

The general content of 3-mixed juice (pear juice mixed with hemp seeds and pumpkin seeds) was analyzed in accordance with the Korean Food Standards Codex. The moisture content was analyzed by a 105 °C atmospheric pressure heating and drying method, crude protein content was determined by the micro-Kjeldahl method using an automatic nitrogen analyzer (Kjeltec Auto 2300, Foss, Hilleroed, Denmark), crude fat content by the acid hydrolysis method, and crude ash content by the direct incineration method.

### Analysis of mineral content

Using a method described in the Korean Food Standards Codex, mineral content was analyzed by mixing 1 g of pear-seeds mixed juice and 10 mL of HNO_3_ solution, completely removing the solvent at 190 °C for 35 min while increasing the temperature by using a microwave (Multiwave 3000, Anton Paar Gmbh, Graz, Austria), and cooling the sample before analysis. Spectrophotometry was performed using an ICP-OES (Optima 8300, PerkinElmer, Waltham, MA, USA).

### Analysis of vitamin content

Vitamin C was analyzed as described by Furusawa (2001), while vitamin E was determined as defined in the Korean Food Standards Codex using high-performance liquid chromatography (Agilent 1200, Agilent Co., Santa Clara, CA, USA).

### Analysis of total polyphenol content (TPC), total flavonoid content (TFC) and total proanthocyanidin content (TPCC)

TPC, TFC and TPCC, which are closely correlated with antioxidant activity, were measured as described by Folin and Denis (1912), Davis (1947) and Baoshan et al. (1998), respectively. The calibration curve of TPC, TFC and TPCC was drawn using gallic acid, naringin and grape seeds oligomeric proanthocyanidins, respectively.

### Analysis of procyanidin B2 content

To measure procyanidin B2, juice samples were prepared as described by Adamson et al. (1999) and high-performance liquid chromatography (Waters e2695, Waters Co., Milford, MA, USA) analyses were performed according to a modified standard process (da Silva Padilha et al., 2017). The column used was an Agilent ODS special analysis column (250 × 4.6 mm, 5 µm). The column temperature was maintained at 35 °C and the injection volume was 10 µL. Juice sample previously diluted to phase A and filtered using 0.22 µm membrane and the flow rate was 1 mL/min. The gradient used in the separation was 0–23 min: 50% B; 23–23.1 min: 0% B; 23.1–30 min: 0% B, where solvent A was 0.1% (v/v) phosphoric acid and solvent B was acetonitrile. The detection of procyanidin B2 was performed at 220 nm using a UV detector (Waters2489, Waters Co., Milford, MA, USA).

### Preparation of water extract of pear-seeds mixed juice

To measure the IC_50_ of ACE inhibitory activity in pear-seeds mixed juice and NO formation potential in vascular endothelial cells, as well as to perform antioxidant content analysis and activity experiment, water extract was prepared from pear-seeds mixed juice by mixing 300 g of pear-seeds mixed juice, 150 mL of *n*-hexane, and 450 mL of distilled water, followed by shaking for 1 h, transfer into a separatory funnel, and incubation for 2 h. Each of the two separated layers was transferred into an Erlenmeyer flask. The collected distilled water was completely dried using a freeze dryer.

### Cell culture

EA.hy926 cells (American Type Culture Collection, Manassas, VA, USA) were cultured in the DMEM containing 10% fetal bovine serum, 2 mM glutamine, and 100 µg/mL penicillin–streptomycin in an incubator (MCO-15AC, SANYO Electric Co., Ltd., Gunma, Japan) maintained at 5% CO_2_ and 95% or higher humidity at 37 °C.

### MTT assay

According to the method of Hansen et al. (1989), the cytotoxicity of EA.hy926 cells in pear seeds juice was measured using the MTT assay. Viability of cells was quantified as a percentage compared to the control (absorbance of treated cells/absorbance of blank × 100) and dose response curves were developed.

### Nitric oxide assay

The amount of NO generated from EA.hy926 cells was measured in the form of NO_2_^−^ in the cell culture solution using the spectrophotometric method (Miranda et al., 2001). EA.hy926 cells were adjusted to 1 × 10^5^ cell/well using DMEM, inoculated into 24-well plates, and cultured in a humidified incubator (MCO-15AC, SANYO Electric Co., Ltd., Gunma, Japan) at 37 °C and in 5% CO_2_. Next, 100 μg/mL water extracts of pear-only juice and pear-seeds mixed juice were pretreated in cells, and the cells were cultured for 24 h. The cell culture supernatant and the same volume of Griess reagent were mixed and reacted for 10 min in 96-well plates, after which their absorbance was measured with a spectrophotometer (Multiskan GO) at 540 nm.

### Analysis of DNA damage by hydroxyl radical using electrophoresis

The oxidation of DNA exposed to the hydroxyl radical created by Fenton’s reaction was performed in accordance with an existing experimental method (Oyaizu, 1986). One hundred microliters of the DNA reaction mixture was prepared by adding 100 µg/mL of the water extracts of juice, 3, 3.5 and 4 mM of FeSO_4_, 2 mM of H_2_O_2_ and 50 µg/mL final concentration of genomic DNA in the same order. The mixture was reacted for 30 min at ambient temperature, and the reaction was stopped by adding 10 mM EDTA. The 20 µL reacted mixture containing 1 µg DNA was electrophoresed in a 1% agarose gel at 100 V for 30 min. The gel was dyed with Midori Green and observed under UV light using the DAVINCH-Chemi Imaging System (CAS-400SM, Corebiosystem, Seoul, Korea).

### DPPH radical scavenging activity

The DPPH radical scavenging activity was measured by modifying Blois’s method (1958). Gallic acid was used as the negative control group.

### Ferric reducing antioxidant power (FRAP) assay

The reducing antioxidant power was measured by the FRAP assay as described by Benzie and Strain (1996). With FeSO_4_, the calibration curve was drawn and each 1 g of sample was expressed in FeSO_4_ µmol (µM FeSO_4_/g).

### Statistical analysis

All analyses were performed at least 3 times and expressed as means and standard deviations (mean ± SD). For a significant difference in the mean, this study employed Duncan’s multiple comparisons in one-way analysis of variance by using SPSS (version 20.0, SPSS, Inc., Chicago, IL, USA). The mean between the two experimental groups was analyzed by Turkey’s multiple comparison test, and *p* values of *p *< 0.05 were considered significant.

## Results and discussion

### ACE inhibitory activity of individual fruits and seeds

Twenty-eight fruits and seeds were selected, and antihypertensive materials were screened in an ACE inhibitory activity assay (Table 1). Among the 13 fruits, pear (17.22%) showed the highest activity level, followed by nectarines (16.93%) and melons (8.19%). Evaluation of seeds’ ACE inhibitory (ACEI) activity showed that among 15 seeds, lentils (89.74%) showed the highest level of activity, followed by common buckwheat (85.36%) and black soybeans (84.07%). While fruits showed a low level of ACEI activity overall, seeds showed a high level of ACEI activity; particularly, legumes such as black soybeans, mung bean, and lentils showed high levels of ACEI activity. Similarly, according to a report of plant food-derived ACE inhibitory peptides, 12 plant food-derived peptides showed a high level of ACEI activity, 7 of which were seeds (soybean, mung bean, sunflower, rice, corn, wheat, and buckwheat) (Guang and Phillips, 2009). These findings demonstrate that proteins contained in seeds are potential sources as ACE inhibitory agents. In contrast, there is a lack of research on the ACE inhibitory properties of fruits. To develop fruit–seed combinations that which can supplement fruits’ low level of ACE inhibitory activity, this study examined the ACE inhibitory activity in fruit–seed combinations.

**Table 1:** ACE inhibitory activity of individual fruits and seeds

Common name	Scientific name	ACE inhibition rate (%)
Fruits		
Apple	*Malus pumila*	3.48 ± 0.85^a^
Aronia	*Aronia melanocarpa*	8.77 ± 2.50^b^
Banana	*Musa paradisiaca*	ND
Grape	*Vitis vinifera*	8.61 ± 0.46^b^
Grapefruit	*Citrus paradisi*	ND
Nectarine	*Prunus persica var.* *nuclpersica*	16.93 ± 1.43^c^
Melon	*Cucumis melo*	8.19 ± 0.02^b^
Orange	*Citrus sinensis*	1.84 ± 0.70^a^
Oriental melon	*Cucumis melo var. makuwa*	ND
Peach	*Prunus persica*	4.78 ± 1.21^a^
Pear	*Pyrus pyrifolia*	17.22 ± 1.18^c^
Pineapple	*Ananas comosus*	1.87 ± 1.65^a^
Plum	*Prunus salicina*	ND
Seeds		
Black soybean	*Glycine max*	84.07 ± 1.31^j^
Common buckwheat	*Fagopyrum esculentum*	85.36 ± 0.84^jk^
Evening primrose seed	*Oenothera erythrosepala*	39.93 ± 1.78^ef^
Hemp seed	*Cannabis sativa*	48.93 ± 0.94^g^
Job’s tear	*Coix lacryma*-*jobi*	41.83 ± 1.31^f^
Lentils	*Lens culinaris*	89.74 ± 0.56^k^
Mung bean	*Phaseolus radiatus*	76.72 ± 1.08^i^
Oat	*Avena sativa*	26.01 ± 1.90^d^
Peanut	*Arachis hypogaea*	21.14 ± 1.79^cd^
Perilla seed	*Perilla frutescens*	18.74 ± 1.84^c^
Pumpkin seed	*Cucurbita moschata*	72.91 ± 0.73^hi^
Safflower seed	*Carthamus tinctorius*	ND
Sesame seed	*Sesamum indicum*	68.30 ± 0.58^h^
Sunflower seed	*Helianthus annuus*	36.03 ± 0.93^e^
Tartary buckwheat	*Fagopyrum tataricum*	73.33 ± 0.48^hi^

In vitro ACE inhibitory activities of fruits and seeds. Data are means ± standard deviations of three experiments

ND, not detected

The difference superscript letter indicates a significant difference from each other (*p* < 0.05) by Duncan’s multiple test

### ACE inhibitory activity in fruit–seed combinations

To supplement the fruits’ low level of ACE inhibitory activity, 15 seeds were extracted along with fruits at a ratio of 7–3 (fruits to seeds), and the ACEI activity for a total of 90 combinations was examined and expressed as the expected value and observed value (Table 2). Synergistic, additive and antagonistic effects were defined as described by Wang et al. (2011). The observed value for 90 combinations was compared with the expected value, which was expressed as the sum of ACE inhibition rates of extracts diluted in 70% (seeds) and 30% (fruits). Accordingly, all interactions were compared using the same total mass. For instance, the ACEI activity (observed value) of a 1.0 g mixture was compared with the sum of ACE inhibition rates (expected value) of 0.7 g fruits and 0.3 g seeds. When the observed value was higher than the expected value, it was determined that a synergistic interaction occurred in the mixture. The opposite result, where the observed value was significantly lower than the expected value, was defined as an antagonistic interaction. No significant difference between the two values indicated an additive interaction. Pear showed a synergistic interaction in 8 combinations. In addition, peaches showed a synergetic interaction in 7 combinations, nectarines in 6, aronia in 7, melons in 8, and grapes in 7. Furthermore, the correlation varied depending on the food category of the seed mixed with the fruit. When legumes such as black soybeans, mung beans, and lentils were mixed with fruits, black soybeans showed an antagonistic interaction with all fruits, only one combination of mung beans and melons showed a synergistic interaction, and legumes showed an antagonistic interaction with nearly all fruits. Additionally, in grains such as oats, tartary buckwheat, common buckwheat, and job’s tears, only one combination of aronia and job’s tears showed a synergistic effect, while most other grains showed additive or antagonistic interactions. In contrast, most nut seeds (evening primrose seed, perilla seed, sesame seed, sunflower seed, hemp seed, pumpkin seed, and safflower seed) showed higher levels of ACEI activity when mixed with fruits. Similarly, a previous study showed that combinations of different food categories had a high synergetic interaction for antioxidant activity, and combinations of different food categories showed a higher synergetic interaction than those in the same food category (Wang et al., 2011). Wang et al. (2011) reported that combinations of fruits and legumes showed the highest synergistic interactions for antioxidant activity such as DPPH radical scavenging activity and reducing activity, which differed from the findings of this study in which that most combinations of fruits and legumes showed an antagonistic interaction for ACE inhibitory activity. Nevertheless, a previous report found that combinations of dried fruits and nuts had a higher level of antioxidant activity than when consumed alone (Jacobs and Steffen, 2003). Accordingly, the results of the present study suggest that consuming combinations of fruits and nut seeds can further enhance their ACE inhibitory activities.

**Table 2:** ACE inhibitory activity in fruit–seed combinations

7:3	Observed value	Expected value	Result	7:3	Observed value	Expected value	Result
AR + BS	70.78 ± 0.48*	94.32 ± 7.44	An	GR + BS	79.67 ± 4.57*	96.90 ± 2.54	An
AR + BW	83.3 ± 0.25*	92.07 ± 3.84	An	GR + BW	85.77 ± 2.88	94.65 ± 4.01	Ad
AR + EP	48.03 ± 2.15*	16.35 ± 8.50	Sy	GR + EP	51.33 ± 4.79*	18.93 ± 3.22	Sy
AR + HP	65.56 ± 2.06*	21.13 ± 5.99	Sy	GR + HP	69.30 ± 5.59*	23.71 ± 8.83	Sy
AR + JT	30.63 ± 3.45*	15.31 ± 2.56	Sy	GR + JT	16.68 ± 4.53	17.90 ± 7.26	Ad
AR + LT	72.91 ± 1.32*	90.16 ± 8.02	An	GR + LT	82.49 ± 0.58*	92.74 ± 2.78	An
AR + MB	29.65 ± 4.58	38.97 ± 8.64	Ad	GR + MB	28.74 ± 1.92*	41.55 ± 3.41	An
AR + OT	20.40 ± 4.14	18.12 ± 9.94	Ad	GR + OT	17.98 ± 1.47	20.70 ± 4.67	Ad
AR + PN	27.28 ± 3.15	17.26 ± 9.05	Ad	GR + PN	31.11 ± 2.13*	19.84 ± 3.95	Sy
AR + PS	46.67 ± 1.84*	14.66 ± 10.76	Sy	GR + PS	45.95 ± 4.28*	17.24 ± 5.55	Sy
AR + PK	46.01 ± 0.95*	23.38 ± 2.16	Sy	GR + PK	63.36 ± 8.77*	25.96 ± 6.05	Sy
AR + SF	15.97 ± 2.29	5.29 ± 8.43	Ad	GR + SF	25.74 ± 1.81	7.87 ± 3.15	Ad
AR + SS	32.22 ± 1.19*	28.59 ± 0.76	Sy	GR + SS	48.53 ± 4.47*	31.17 ± 5.13	Sy
AR + SU	35.83 ± 0.82*	21.30 ± 1.96	Sy	GR + SU	64.82 ± 2.57*	23.88 ± 4.42	Sy
AR + TB	79.92 ± 1.57	87.29 ± 8.57	Ad	GR + TB	83.94 ± 0.58	89.87 ± 3.48	Ad
NT + BS	77.98 ± 0.57*	101.48 ± 8.82	An	ML + BS	87.89 ± 0.50*	92.94 ± 0.56	An
NT + BW	84.84 ± 0.72	99.23 ± 10.75	Ad	ML + BW	82.50 ± 2.01	90.69 ± 1.69	Ad
NT + EP	42.80 ± 3.04*	23.50 ± 2.49	Sy	ML + EP	26.51 ± 2.44*	14.97 ± 2.68	Sy
NT + HP	30.81 ± 2.76	28.28 ± 4.46	Ad	ML + HP	80.87 ± 2.50*	19.75 ± 5.81	Sy
NT + JT	9.57 ± 1.14	22.47 ± 10.53	Ad	ML + JT	10.26 ± 1.67	13.94 ± 5.43	Ad
NT + LT	72.62 ± 1.42*	97.32 ± 8.16	An	ML + LT	88.51 ± 0.90	88.78 ± 1.58	Ad
NT + MB	23.86 ± 1.52*	46.13 ± 7.22	An	ML + MB	50.31 ± 3.75*	37.59 ± 0.46	Sy
NT + OT	13.19 ± 3.20	25.28 ± 9.09	Ad	ML + OT	16.18 ± 0.23	16.74 ± 2.88	Ad
NT + PN	17.64 ± 3.26	24.42 ± 9.41	Ad	ML + PN	32.06 ± 8.52	15.88 ± 1.53	Ad
NT + PS	39.11 ± 0.16*	21.81 ± 1.11	Sy	ML + PS	80.44 ± 2.34*	13.28 ± 3.32	Sy
NT + PK	51.39 ± 3.25*	30.54 ± 1.34	Sy	ML + PK	85.33 ± 7.09*	22.00 ± 3.59	Sy
NT + SF	42.48 ± 3.68*	12.45 ± 7.65	Sy	ML + SF	51.50 ± 3.56*	3.91 ± 2.48	Sy
NT + SS	41.54 ± 2.20*	35.75 ± 2.61	Sy	ML + SS	58.93 ± 7.03*	27.21 ± 4.05	Sy
NT + SU	70.49 ± 1.75*	28.46 ± 9.80	Sy	ML + SU	76.92 ± 2.07*	19.92 ± 1.88	Sy
NT + TB	83.82 ± 0.22*	94.45 ± 9.14	An	ML + TB	77.07 ± 6.72	85.91 ± 1.14	Ad
PC + BS	76.14 ± 3.25*	93.64 ± 0.81	An	PE + BS	66.84 ± 1.94*	90.49 ± 1.20	An
PC + BW	84.36 ± 1.77	91.39 ± 2.52	Ad	PE + BW	79.89 ± 0.78*	88.23 ± 2.77	An
PC + EP	43.80 ± 5.62*	15.67 ± 2.47	Sy	PE + EP	40.82 ± 2.76*	12.51 ± 2.64	Sy
PC + HP	58.48 ± 8.05*	20.45 ± 7.09	Sy	PE + HP	76.46 ± 4.12*	17.29 ± 7.48	Sy
PC + JT	26.14 ± 7.06	14.63 ± 6.06	Ad	PE + JT	18.14 ± 0.71*	11.48 ± 6.37	Sy
PC + LT	77.50 ± 2.06*	89.48 ± 1.54	An	PE + LT	71.50 ± 1.43*	86.32 ± 1.81	An
PC + MB	25.89 ± 1.15	38.29 ± 2.84	Ad	PE + MB	19.05 ± 2.46*	35.13 ± 2.97	An
PC + OT	24.23 ± 3.85	17.44 ± 3.40	Ad	PE + OT	12.24 ± 3.69	14.28 ± 3.72	Ad
PC + PN	22.65 ± 9.03	16.58 ± 2.37	Ad	PE + PN	15.86 ± 3.36	13.42 ± 2.74	Ad
PC + PS	40.50 ± 5.93*	13.97 ± 4.12	Sy	PE + PS	36.49 ± 2.32*	10.82 ± 4.47	Sy
PC + PK	36.97 ± 2.29*	22.70 ± 4.53	Sy	PE + PK	63.70 ± 0.50*	19.54 ± 4.90	Sy
PC + SF	19.49 ± 2.77*	4.61 ± 2.31	Sy	PE + SF	18.44 ± 3.12*	1.46 ± 2.50	Sy
PC + SS	45.44 ± 7.10*	27.91 ± 4.25	Sy	PE + SS	36.56 ± 2.15*	24.75 ± 4.49	Sy
PC + SU	46.00 ± 6.12*	20.62 ± 2.82	Sy	PE + SU	40.15 ± 0.22*	17.47 ± 3.20	Sy
PC + TB	78.94 ± 3.69*	86.61 ± 1.89	An	PE + TB	78.25 ± 0.66*	83.45 ± 2.26	An

AR, aronia; Gr, grape; NT, nectarine; ML, melon; PC, peach; PE, pear; BS, black soybean; BW, common buckwheat; EP, evening primrose seed; HP, hemp seed; JT, job’s tear, LT, lentils; MB, mung bean; OT, oats; PN, peanut; PS, perilla seed; PK, pumpkin seed; SF, safflower seed; SS, sesame seed; SU, sunflower seed; TB, tartary buckwheat; Sy, synergistic interaction; Ad, additive interaction; An, antagonistic interaction

The asterisk indicates a significant difference between observed value and expected value (*p* < 0.05) by Turkey *t* test

### ACE inhibitory activity in 3-mixed composition

Table 3 shows the ACEI activity changes for 114 combinations (3-mixed), which combined 1 fruit and 2 seeds from the fruit–seed combinations showing synergetic interactions for ACEI activity. Most 3-mixed combinations were similar to those of 2-mixed combinations or showed decreased ACEI activity. However, the 3-mixed juice of pear, hemp seeds and pumpkin seeds increased to 95.35% in ACE inhibitory activity and showed a considerable level of synergetic interaction. When hemp seeds (48.93 ± 0.94%) and pumpkin seeds (72.91 ± 0.73%) were individually added to pear (17.22 ± 1.18%), the pear-hemp seed juice showed 76.46% ACE inhibitory activity, which is 4.42-fold higher than the expected value, while pear-pumpkin seed juice showed 63.70%, which is 3.26-fold higher than the expected value. In comparison, the pear-hemp seed-pumpkin seed juice ACE inhibitory activity was 1.25- and 1.50-fold higher than the 2-mixed combinations, respectively; the 3-mixed combination showed a strong synergetic interaction. According to Girgih et al. (2014), protein hydrolysates contained in hemp seeds showed 70% ACE inhibitory activity and 35% renin inhibitory activity in vitro and were reported to have strong BP lowering effect. Although hemp seed proteins that did not undergo hydrolysis showed a low activity compare with hydrolysates, they also significantly lowered the BP of spontaneously hypertensive rats and showed ACE and renin inhibitory activity in blood plasma. Similarly, in recent study, low molecular weight peptides of hemp seed protein hydrolysates were investigated on ACE inhibition activity (Orio et al., 2017). The main reason for this choice was that law molecular weight peptide was stable towards stomach proteases, efficient absorption at intestinal level and especially ACE-inhibitors peptides are generally short chained. As a result, among of the tested bioactive peptides, GVLY peptide exhibited the highest ACE inhibition activity. Meanwhile, the vasodilating effect of pumpkin seeds was established by Chelliah et al. (2018) in recent study. Chelliah et al. (2018) reported that cucurbitin protein belonging to 11S globulin family from pumpkin seeds shows higher vasodilating effect than 11S globulin from white sesame and amandin protein from almond. Furthermore, the findings that pumpkin seed oils showed a good interaction with hypertension drugs of felodipine and captopril and had potential BP lowering activity are consistent with the results of this study, in which pumpkin seed combinations showed a higher level of synergetic interactions (AL Zuhair et al., 2000). Accordingly, this study examined the effect of lowering BP and antioxidant activity of 3-mixed juice, in which pear was mixed with hemp seeds and pumpkin seeds at a ratio of 7–3.

**Table 3:** ACE inhibitory activity in 3-mixed composition

3-Mixed composition	ACE inhibition rate (%)	3-Mixed composition	ACE inhibition rate (%)	3-Mixed composition	ACE inhibition rate (%)
AR + EP + PS	31.63 ± 1.71	GR + SS + PK	61.25 ± 2.37	ML + SS + PK	34.18 ± 1.35
AR + EP + JT	17.39 ± 0.31	GR + SU + HP	29.53 ± 2.14	ML + SS + SF	28.92 ± 3.80
AR + EP + SS	26.48 ± 3.86	GR + SU + PK	53.60 ± 1.58	ML + SU + HP	66.76 ± 4.75
AR + EP + SU	33.44 ± 1.34	GR + HP + PK	32.69 ± 3.38	ML + SU + PK	60.57 ± 6.86
AR + EP + HP	23.54 ± 5.29	NT + EP + PS	52.49 ± 8.85	ML + SU + SF	16.35 ± 9.44
AR + EP + PK	37.14 ± 4.31	NT + EP + SS	54.35 ± 14.24	ML + HP + PK	48.20 ± 3.88
AR + PS + JT	21.69 ± 10.15	NT + EP + SU	74.57 ± 6.09	ML + HP + SF	17.78 ± 4.75
AR + PS + SS	16.16 ± 6.31	NT + EP + HP	41.47 ± 11.65	ML + PK + SF	5.35 ± 3.70
AR + PS + SU	20.81 ± 4.57	NT + EP + PK	66.22 ± 6.87	PC + EP + PS	36.01 ± 10.44
AR + PS + HP	28.13 ± 5.16	NT + EP + SF	58.93 ± 7.81	PC + EP + SS	33.08 ± 9.41
AR + PS + PK	16.18 ± 4.43	NT + PS + SS	42.52 ± 9.57	PC + EP + SU	48.70 ± 7.87
AR + JT + SS	15.23 ± 3.68	NT + PS + SU	64.80 ± 4.08	PC + EP + HP	44.21 ± 6.30
AR + JT + SU	14.94 ± 1.60	NT + PS + HP	33.67 ± 10.96	PC + EP + PK	42.63 ± 4.46
AR + JT + HP	9.02 ± 1.14	NT + PS + PK	59.00 ± 4.77	PC + PS + SS	34.45 ± 10.49
AR + JT + PK	11.61 ± 5.64	NT + PS + SF	55.14 ± 7.49	PC + PS + SU	47.91 ± 6.97
AR + SS + SU	15.37 ± 1.10	NT + SS + SU	56.40 ± 7.42	PC + PS + HP	61.69 ± 5.60
AR + SS + HP	17.90 ± 9.66	NT + SS + HP	42.25 ± 10.76	PC + PS + PK	45.62 ± 13.79
AR + SS + PK	29.21 ± 1.95	NT + SS + PK	49.41 ± 7.41	PC + SS + SU	42.18 ± 9.69
AR + SU + HP	14.56 ± 2.80	NT + SS + SF	51.35 ± 9.16	PC + SS + HP	54.79 ± 9.01
AR + SU + PK	15.41 ± 1.50	NT + SU + HP	37.54 ± 10.05	PC + SS + PK	48.64 ± 5.84
AR + HP + PK	12.16 ± 4.00	NT + SU + PK	67.64 ± 10.39	PC + SU + HP	59.95 ± 8.01
GR + EP + PN	29.10 ± 1.71	NT + SU + SF	63.09 ± 7.16	PC + SU + PK	45.76 ± 10.57
GR + EP + PS	45.90 ± 3.64	NT + HP + PK	27.15 ± 4.24	PC + HP + PK	36.68 ± 9.50
GR + EP + SS	42.08 ± 1.31	NT + HP + SF	35.26 ± 11.67	PE + EP + PS	27.55 ± 4.78
GR + EP + SU	60.04 ± 3.41	NT + PK + SF	55.55 ± 11.01	PE + EP + SS	21.37 ± 1.59
GR + EP + HP	49.87 ± 9.16	ML + MB + PS	17.47 ± 1.44	PE + EP + SU	47.48 ± 2.86
GR + EP + PK	54.82 ± 2.75	ML + MB + SS	32.45 ± 7.27	PE + EP + HP	44.40 ± 2.01
GR + PN + PS	37.46 ± 2.91	ML + MB + SU	54.89 ± 3.70	PE + EP + PK	41.91 ± 3.74
GR + PN + SS	28.75 ± 1.85	ML + MB + HP	66.75 ± 7.81	PE + PS + SS	32.24 ± 5.15
GR + PN + SU	37.51 ± 8.69	ML + MB + PK	21.13 ± 2.90	PE + PS + SU	56.24 ± 1.94
GR + PN + HP	30.89 ± 1.23	ML + MB + SF	47.51 ± 2.01	PE + PS + HP	59.01 ± 3.01
GR + PN + PK	25.50 ± 1.80	ML + PS + SS	65.90 ± 3.26	PE + PS + PK	50.52 ± 0.74
GR + PS + SS	33.17 ± 2.54	ML + PS + SU	44.73 ± 1.16	PE + SS + SU	39.72 ± 3.09
GR + PS + SU	54.90 ± 0.69	ML + PS + HP	51.68 ± 5.39	PE + SS + HP	86.44 ± 2.60
GR + PS + HP	31.69 ± 1.65	ML + PS + PK	59.48 ± 5.05	PE + SS + PK	52.26 ± 5.88
GR + PS + PK	48.25 ± 7.44	ML + PS + SF	7.20 ± 4.05	PE + SU + HP	67.64 ± 2.02
GR + SS + SU	53.58 ± 2.43	ML + SS + SU	42.73 ± 4.35	PE + SU + PK	80.61 ± 3.39
GR + SS + HP	56.11 ± 3.11	ML + SS + HP	67.75 ± 4.56	PE + HP + PK	95.35 ± 0.13

Data are means ± standard deviations of three experiments

AR, aronia; BS, black soybean; BW, common buckwheat; EP, evening primrose seed; HP, hemp seed; JT, job’s tear; LT, lentils; MB, mung bean; OT, oats; PN, peanut; PS, perilla seed; PK, pumpkin seed; SF, safflower seed; SS, sesame seed; SU, sunflower seed; TB, tartary buckwheat; Sy, synergistic interaction; Ad, additive interaction; An, antagonistic interaction

### Nutritional composition of juice samples

Pear is the third most widely produced fruit in Korea, following citrus fruits and apples, and known to have a high fiber content, which reduces the occurrence of CVDs and cancer, as well as high levels of arbutin, making it effective for treating cough and inflammation (Tanrıöven and Ekşi, 2005). However, while the consumption of citrus fruits and apples as juice is quite high in proportion to their production, pear juice only accounts for 0.1% of the total consumption. This is because pear juice has a low acid flavor and insipid flavor and because its pulp density is too high; consumer needs can be satisfied using different processing methods. Accordingly, this study mixed and extracted pear, which is produced abundantly and has a variety of physiological functional activities, with hemp seeds and pumpkin seeds; the nutrient contents of pear juice and 3-mixed juice were compared (Table 4). In pear juice, moisture accounted for 88.18% of the content, followed by carbohydrate (10.88%), protein (0.43%), crude ash (0.25%), and fat (0.17%). In 3-mixed juice, moisture accounted for 66.97% of the content, which is 1.32-fold lower than in pear juice, its protein content was 21.98-fold higher, fat content was 69.65-fold higher, and crude ash content was 6.60-fold higher. When pear was mixed and extracted with seeds, the moisture content decreased, while the protein, fat, and crude ash contents increased; as a result, the mineral contents increased for calcium, magnesium, and potassium by 12.38-, 24.85-, and 2.74-fold, respectively. Although iron was not detected in pear-only juice, 2.07 mg/100 g of iron was found because of the added seeds in 3-mixed juice. Potassium is among the most important minerals affecting hypertension, and a diet low in potassium and high in sodium is known to be directly correlated with hypertension (Aburto et al., 2013; Mokotedi et al., 2018). In addition, magnesium eases vascular muscles and maintains low BP (Kass et al., 2012). Accordingly, supplementation with the BP-lowering minerals that are lacking in pear by adding seeds may enable 3-mixed juice to be developed as an antihypertensive beverage. Additionally, the vitamin C and E contents of 3-mixed juice, to which seeds were added, were 1.40- and 56-fold higher than those of pear juice, respectively. Both vitamins C and E are known to play a positive role in hypertension prevention and treatment. Vitamin C inhibits the dissolution of nitric oxide in the blood vessels and affects endothelium-dependent vasodilation (May, 2000), while vitamin E increases the activity of nitric oxide synthases, a nitric oxide synthesis enzyme; thus, these vitamins lower BP (Newaz et al., 1999). These findings suggest that adding seeds to pear juice can supplement the insufficient minerals and vitamins and produce a higher level of synergetic interaction. In addition, analysis of the contents of polyphenolic compounds, which are known to show physiological activity in most plants, found that the polyphenol content of 3-mixed juice was 10.62-fold higher than that of pear-only juice, while its flavonoid content was 10.20-fold higher than that of pear-only juice. Flavonoids are the largest group among polyphenolic compounds, and two phenyl rings are combined with 3 carbons, which are in a closed pyran ring. Flavonoids are widespread in plants and show various physiological activities. Apigenin, butein, epicatechin, luteolin, and quercetin are known to have a higher level of ACE inhibitory activity (Balasuriya and Rupasinghe, 2011). Within flavonoids, procyanidins and proanthocyanidins known as condensed tannin has been reported to exhibit various bioactive properties such as antioxidant, anticancer and cardioprotective activities so these compounds have attracted increased attention in the fields of nutrition, health, and medicine largely (Rue et al., 2018). In this study, total proanthocyanidin and procyanidin B2 contents of 3-mixed juice were investigated. As a result, it was found that 3-mixed juice contained 11.44-fold higher proanthocyanidin and 10.89-fold higher procyanidin B2 than pear juice. These results significantly correlate with polyphenol and flavonoid contents. Hence, the synergetic interaction for ACE inhibitory activity observed after adding seeds to pear juice is thought to be closely correlated with increased polyphenol, flavonoid, proanthocyanidin and procyandin B2 contents.

**Table 4:** Nutrition composition of 3-mixed juice

Composition	Unit	Pear juice	3-Mixed juice
General compositions			
Carbohydrates	g/100 g	10.88 ± 0.17^a^	10.08 ± 0.20^a^
Crude protein	g/100 g	0.43 ± 0.01^a^	9.45 ± 0.05^b^
Crude lipid	g/100 g	0.17 ± 0.03^a^	11.84 ± 0.03^b^
Crude ash	g/100 g	0.25 ± 0.03^a^	1.65 ± 0.05^b^
Moisture	g/100 g	88.18 ± 0.02^a^	66.97 ± 0.23^b^
Calorie	kcal/100 g	46.74 ± 0.44^a^	184.74 ± 1.14^b^
Mineral			
Calcium	mg/100 g	0.90 ± 0.14^a^	11.14 ± 0.42^b^
Magnesium	mg/100 g	5.96 ± 0.10^a^	148.09 ± 0.72^b^
Potassium	mg/100 g	119.77 ± 0.63^a^	328.45 ± 0.11^b^
Iron	mg/100 g	ND^a^	2.07 ± 0.04^b^
Vitamin			
Vitamin C	mg/100 g	5.3 ± 0.92^a^	7.4 ± 0.72^b^
Vitamin E	mg α-TE/100 g	0.03 ± 0.00^a^	1.68 ± 0.00^b^
Phenolic compounds			
TPC	mg GAE/100 g	80.11 ± 4.80^a^	850.52 ± 5.39^b^
TFC	mg NE/100 g	22.26 ± 0.78^a^	226.97 ± 5.93^b^
TPCC	mg/100 g	4.41 ± 0.89^a^	50.47 ± 1.94^b^
Procyanidin B2	mg/100 g	4.35 ± 0.01^a^	47.36 ± 0.02^b^

Data are means ± standard deviations of at least three replicates of juice samples

ND, not detected; TPC, total polyphenolic compound content, expressed in mg of GAE (gallic acid equivalents)/100 g of sample; TFC, total flavonoid content, expressed in mg of NE (naringin equivalents)/100 g of sample; TPCC, total proanthocyanidin content

The difference superscript letter indicates a significant difference from each other (*p* < 0.05) by Duncan’s multiple test. 3-mixed juice, pear juice mixed with pumpkin seeds and hempseeds

### Comparison of IC_50_ values of juice samples

**Fig. 1:**
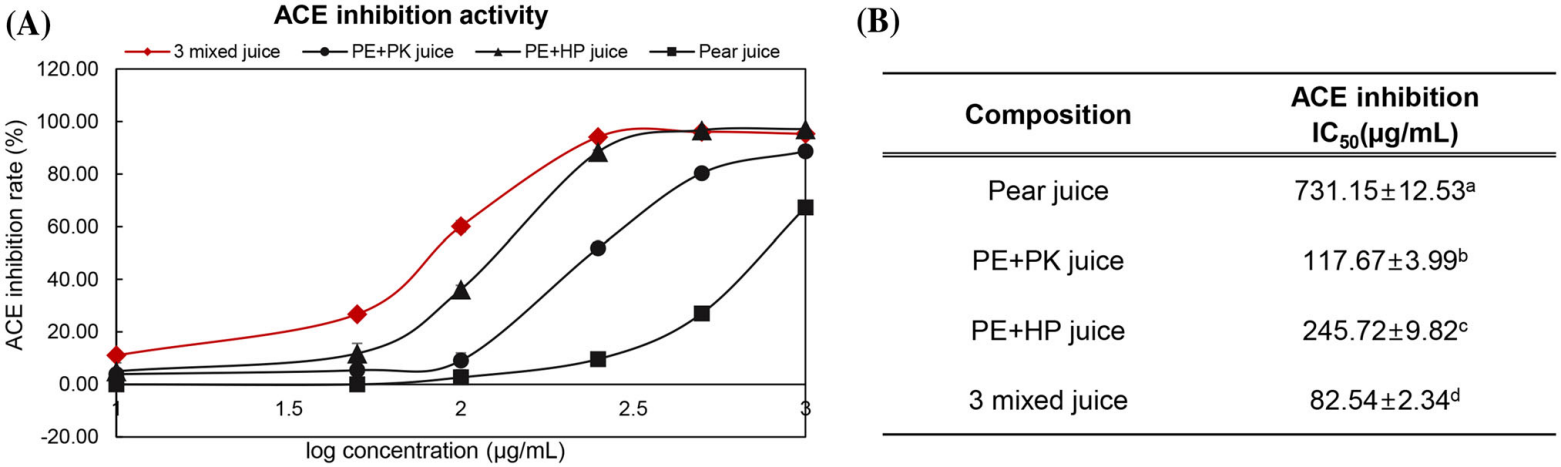
ACE inhibition (IC_50_ values) of juice samples. In vitro ACE inhibitory activities of fruits and seeds. Data are mean ± standard deviation of at least three replicates of juice samples. **A***In vitro* ACE inhibitory activities of juice samples at varying concentrations. **B** The IC_50_ value was determined. The difference letter indicates a significant difference from each other (*p* < 0.05) by Duncan’s multiple test. PE, pear; PK, pumpkin seed; HP, hemp seed

This study also investigated the ACE inhibition IC_50_ values of the 3-mixed juice water extract showing the highest ACEI activity along with other combinations (Fig. 1). As a result, the 3-mixed juice ACE inhibition IC_50_ was 82.54 µg/mL, which was the highest activity, followed by pear-hemp seed juice (245.72 µg/mL), pear-pumpkin seed juice (117.67 µg/mL), and pear-only juice (731.15 µg/mL). When hemp seeds and pumpkin seeds were mixed with pear juice, the ACE inhibitory activity increased by 6.27- and 3.01-fold, respectively. The ACE inhibitory activity of 3-mixed juice was 8.83-fold higher, showing a high level of synergetic interaction. Hemp seed hydrolysates are known to have not only antioxidant activity, but also antihypertensive activity (Girgih et al., 2014). This is because negatively charged amino acids, which can share electrons with reactive oxygen species, are abundant. Furthermore, Girgih et al. (2014) proposed that protein hydrolysis is required to improve antihypertensive efficiency of hemp seeds and, accordingly, a high digestion rate would enhance antihypertensive efficiency of hemp seeds. Hence, proteins contained in seeds are absorbed inside the body, hydrolyzed by various enzymes in the intestines, and can be convert into the peptides with have a high level of ACE inhibitory activity. Thus, the proteins in seeds may play an important role in lowering BP, and seeds can function as a protein supply source for fruits.

### Effect of NO production of juice samples on EA.hy 926 cells

**Fig. 2:**
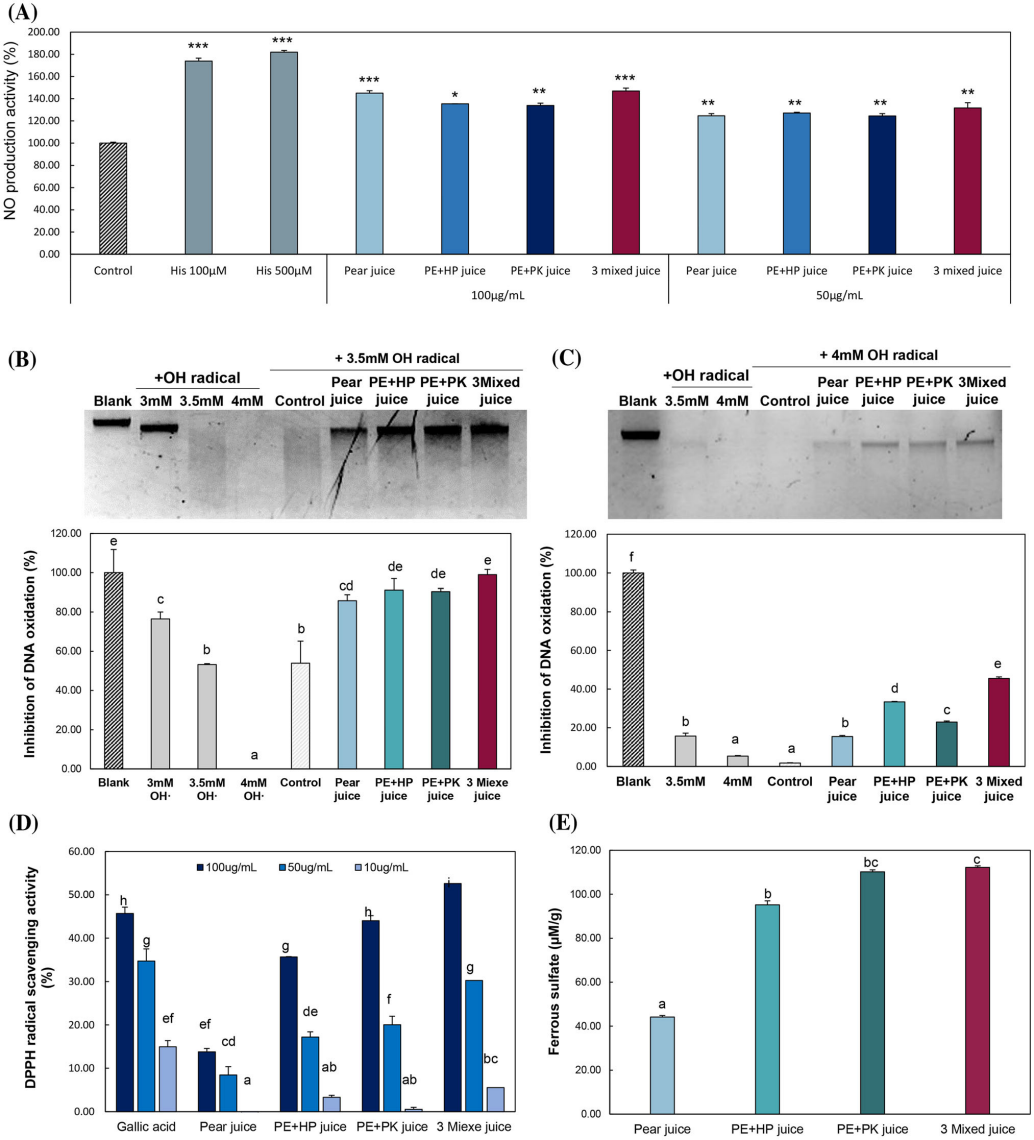
NO production activity on EA.hy926 cells and antioxidant activities of juice samples. Effect of juice samples on NO production (**A**) of EA.hy926 cells. Cells were treated with juice samples and NO production were determined after 24 h. Histamine (His) was used as a positive control for NO production. Data are mean ± standard deviation of at least three replicates of juice samples. The asterisk indicates a significant difference compared with blank (*p* < 0.05). Protective effect of juice samples on DNA oxidative damage induced by OH radical using Fenton’s reaction of 3.5 mM (**B**) and 4 mM (**C**). Genomic DNA purified from EA.hy926 cells. DNA was electrophoresed on a 1% agarose gel for 30 min at 100 V and visualized by UV light after staining with Midori Green. Blank, non-treated DNA; Control, OH radical treated DNA. 2,2-diphenyl-1-picrylhydrazyl (DPPH) radical scavenging activity (**D**) and FRAP assay (**E**) were evaluated in the presence of juice samples. Data are means ± standard deviations of at least three replicates of juice samples. The difference letter indicates a significant difference from each other (*p* < 0.05) by Duncan’s multiple test. PE, pear; PK, pumpkin seed; HP, hemp seed

To examine the effect of pear-seeds mixed juice water extract on NO formation potential, cytotoxic effects were evaluated on EA.hy 926 cells. The MTT assay was conducted to examine the cytotoxicity of pear-seeds mixed juice. Pear-only juice and pear-seeds mixed juice did not show cytotoxicity on EA.hy926 cells below 100 µg/mL. The effects of pear-seeds mixed juice on NO produced from EA.hy926 cells was measured in the cell culture solution by using the Griess assay (Fig. 2A). All pear-only juice and pear-seeds mixed juice produced 1.33–1.47-fold more NO than the blank group. NO production in vascular endothelial cells dilates the blood vessels and plays a direct role in lowering BP. Similarly, hemp seeds have a high level of antihypertensive activity because l-arginine, the precursor of NO, exists in abundance (Girgih et al., 2014). Pumpkin seeds are also known to lower BP because of their abundant l-arginine, which inhibits the production of MDA in a hypertension model induced by N(ω)-nitro-l-arginine methyl ester hydrochloride and facilitates NO production (El-Mosallamy et al., 2012). In addition, the finding that NO production was most active in the 3-mixed juice treatment group was consistent with the vitamin C and E contents. Vitamin C inhibits the dissolution of nitric oxide in blood vessels (May, 2000) and vitamin E increases the activity of nitric oxide synthases, an NO synthesis enzyme, which lowers BP (Newaz et al., 1999). Three-mixed juice contained 1.40- and 56-fold higher vitamin C and E contents, respectively, than pear-only juice, which may facilitate NO production in the blood vessels and enhance the stability of produced NO. Hence, consuming 3-mixed juice may useful in lowering elevated BP by inhibiting ACE activity and promoting NO production in the blood vessels.

### Antioxidant activities of juice samples

The results of analyzing the antioxidant activity in the 3-mixed seed juice water extract are described in Fig. 2. Genomic DNA was exposed to a hydroxyl radical in the Fenton’s reaction, and DNA oxidation was induced (Fig. 2B, C). DNA degradation caused by oxidation occurred more actively as the concentration of products of the Fenton’s reaction increased. However, DNA degradation decreased because of a higher DNA protection effect in all juice water extracts, which were treated with 100 µg/mL. Particularly, 3-mixed juice showed high protection effect similar to that in the blank group, with nearly no DNA degradation from the hydroxyl radical produced at 3.5 mM (Fig. 2B). When DNA was exposed to the hydroxyl radical produced at 4 mM, 94.64% of DNA was degraded. However, only 54.49% of DNA was degraded in the 100 µg/mL 3-mixed juice treatment group, which was 1.74-fold lower, demonstrating a high level of protection effect against the hydroxyl radical (Fig. 2C). Additionally, 2 mixed juice, to which hemp seeds or pumpkin seeds were added, showed DNA degradation rates of 66.59% and 77.05%, respectively, and protection effects that were 1.27- and 1.10-fold higher than those in pear-only juice (84.51%). However, their protection effect was 1.22- and 1.41-fold lower than in the 3-mixed juice. This may be because of the synergetic interaction that arose from 3-mixed juice of pear, hemp seeds, and pumpkin seed. Similarly, analysis of the DPPH radical scavenging activity (Fig. 2D) showed that 100 µg/mL pear-only juice had 13.79% in scavenging activity. In addition, when hemp seeds or pumpkin seeds were added to pear-only juice, the DPPH radical scavenging activity increased by 35.69% and 44.03%, respectively. Notably, however, 3-mixed juice, in which both hemp seeds and pumpkin seeds were added to pear-only juice, showed 52.60% increased scavenging activity, which was higher than the 100 µg/mL gallic acid and produced a greater synergetic interaction. Moreover, according to a FRAP assay (Fig. 2E), pear-seeds juice’s reducing power was highest in 3-mixed juice (112.24 µM FeSO_4_/g) followed by pear-pumpkin seed juice (110.20 µM FeSO_4_/g), pear-hemp seed juice (95.17 µM FeSO_4_/g), and pear juice (44.20 µM FeSO_4_/g). There is a lack of study on antioxidant activity of hemp seed, but according to Girgih et al. (2011) and Tang et al. (2009), it was reported that protein contained abundantly in hemp seeds has an antioxidant activity and its antioxidant activity can be further increased by enzymatic protein hydrolysis. According to the study by Xanthopoulou et al. (2009), roasted pumpkin seed extract showed EC_50_ values of 4.51–6.71 mg/mL for DPPH radical scavenging activity, which were similar to the activity of pumpkin seed juice (44.03%). Thus, the 3-mixed juice can be used to prevent various diseases caused by damage to cells, proteins, and DNA by removing active oxygen species accumulated for many different reasons through its high level of reducing power.

In conclusion, the findings of this study suggest that fruits’ low level of ACE inhibitory activity can be supplemented by mixing with seeds and combination of hemp seed and pumpkin seed was more likely to create synergism on ACE inhibition. In addition, 3-mixed juice (pear, hemp seeds, and pumpkin seeds) may activate NO production in blood vessels, as it contains vitamins C and E as well as *l*-arginine, and may lower BP. Although the mechanism for the correlation between NO and vitamin is still unclear, previous studies has shown that vitamin C lowers blood pressure by inhibiting NO degradation in the blood vessels and vitamin E lowers blood pressure by enhancing the activity of nitric oxide synthases (Engler et al., 2003). Therefore vitamin C and E presented in 3-mixed juice seem to be related to NO stability and NO production, respectively. Furthermore, 3-mixed juice may reduce oxidative stress produced in the blood vessels, and thus may be useful for improving and treating vascular health and function. For these reasons, although this study was focused on ACE inhibition and NO production in EA.hy926 cells, 3-mixed juice can be used as healthy food for the prevention and treatment of hypertension.
